# Maturing conditions of bimetallic nanocomposites as a new factor influencing Au–Ag synergism and impact of Cu(II) and/or Fe(III) on luminescence

**DOI:** 10.1098/rsos.241385

**Published:** 2025-01-22

**Authors:** Veronika Svačinová, Tomáš Pluháček, Martin Petr, Karolina Siskova

**Affiliations:** ^1^ Department of Experimental Physics, Faculty of Science, Palacký University Olomouc, Olomouc, Czech Republic; ^2^ Department of Analytical Chemistry, Faculty of Science, Palacký University Olomouc, Olomouc, Czech Republic; ^3^ RCPTM, Catrin, Palacký University Olomouc, Olomouc, Czech Republic

**Keywords:** bimetallic nanocluster, gold–silver nanocluster, protein template, luminescent nanocomposite, fluorescent nanocomposite, synergistic effect

## Abstract

Gold–silver synergism has been well documented in many scientific works dealing with luminescent nanostructures that are exploitable in biomedical and environmental application. Frequently, the ratio of Au : Ag in synthetic mixtures was varied to influence the extent of Au–Ag synergism of the resulting luminescent gold–silver nanoclusters (GSNCs). However, in our approach, a new step, maturing under differing conditions using the same Au : Ag ratio (5 : 1), has been investigated systematically for the very first time. As referent systems, monometallic gold nanoclusters (AuNCs) and protein treated by the conditions of synthesis and maturing were prepared and investigated. The selected types of maturing conditions led to distinct changes in fluorescence characteristics and, consequently, Au–Ag synergism extent (evaluated as the ratio of fluorescence quantum yields of GSNCs versus AuNCs). The best synergism was obtained for GSNCs matured at 37°C for 2.5 h. The stability of luminescent signal of these GSNCs was tested in the presence of an excess (to 20 mM) of Cu(II) and/or Fe(III) ions (crucial cofactors in living systems). The same metallic ion concentration caused different extents of GSNC luminescence quenching, for which a plausible reasoning is suggested.

## 1. Introduction

In the last decade, luminescent bimetallic gold–silver nanoclusters (GSNCs) have attracted the attention of many researchers since they can possess enhanced luminescent properties in comparison to monometallic Au and/or Ag nanoclusters (NCs) as reviewed in [1]. GSNCs benefit from augmented emission intensity and increased fluorescence quantum yield (FQY), a phenomenon frequently called synergism of gold and silver [1–5]. However, this synergism is not observed always whenever Au and Ag are mixed within a reaction mixture.

GSNCs can be prepared by eco-friendly chemical syntheses quite easily, in similarity to well-known luminescent gold nanoclusters (AuNCs). Indeed, the reduction properties of naturally occurring species, such as nucleotides, peptides and proteins, have been successfully exploited already [2–19]. Hereafter, we focus on GSNCs embedded in protein matrices that create ideal scaffolds for metallic ion entrapment, their consequent reduction with a concomitant growth restriction of the final metallic nanostructures. Stable luminescent noble metal nanostructures are then generated owing to the space limited growth induced by the protein.

GSNCs manifest themselves by large Stokes shifts, no fluorescence intensity changes within a broad pH range (5–12), no fluorescence changes due to ionic strength increase, and no photobleaching [2,6,7] when compared with organic fluorophores and other advanced microfluorescence methods (e.g. reviewed by Praus *et al*. [20]). Thus, due to these unique photoluminescent properties, GSNCs have been successfully applied for quantitative sensing of toxic heavy metal ions (e.g. Hg(II), Pb(II), Cu(II)), anionic species (e.g. cyanides, sulfide, iodide), low molecular weight compounds (e.g. dopamine, cysteine, folic acid) and/or even short peptides (e.g. glutathione (GSH)) already [2–14,21]. Moreover, a strategy for inorganic pyrophosphatase activity monitoring was developed by Zhou *et al*. employing GSNCs [5]. The article of Dutta *et al*. dealt with GSNC exploitation as a theranostic gene delivery vector for HeLa cancer cells [15]. Further biomedical application of GSNCs in cell imaging and temperature sensing was reported in [2]. Another achievement was reached by Sannigrahi *et al*. who detected toxic lead ions not only *in vitro*, but also inside cells [12]. In 2021, Wang and co-workers developed a sensitive ratiometric fluorescent platform for quantitative analysis of α-glucosidase [22]. Very recently, we have developed and applied an inert contrast agent for bimodal imaging containing GSNCs [23]. Thus, GSNC nanocomposites have been successfully applied in many environmental or biomedical research domains so far. It is thus important to understand their formation and to finely tune their luminescent features in order to obtain the best possible optimized results.

The most frequently used protein in GSNC formation is bovine serum albumin (BSA) undoubtedly [3–5,7,10–17,23]. Albeit many authors exploited BSA, dissimilar concentrations of the protein were used, and the other parameters of GSNC syntheses (like temperature, type of heating etc.) also differed significantly in their works leading to incomparable results. Nevertheless, the synergistic effect is pronounced in many of the above-cited articles and experimentally proved by a direct comparison of GSNC luminescent properties with those of monometallic analogues. It can be summarized from the literature study that Au–Ag synergism can be tuned mostly by the Au : Ag ratio [4,5,7,11,14]. There has been even a theoretical study showing that the optical transition of Ag-doped AuNCs is concentration-dependent and the optical transition between highest occupied molecular orbital (HOMO) and lowest unoccupied molecular orbital (LUMO) shifts to the low energy range with increasing Ag content [24]. Moreover, experimental works (e.g. [12]) revealed that by changing the Au : Ag ratio, it is possible to tune the position of GSNC emission maximum towards the infrared (IR) region. Considering the potential application of GSNCs in bio-imaging [23] and, simultaneously, the position of biological optical windows, both factors, synergism and near-IR emission, would be very beneficial. Hence, motivated by this idea, we tried to prepare GSNCs embedded in BSA with the aim to improve luminescent properties of our previous AuNCs [25,26].

In the present work, we show that besides varying the Au : Ag ratio in the reaction mixture (as known from the literature), there is another possibility for tuning Au–Ag synergism while keeping the same molar ratio of Au : Ag in the reaction mixture. We introduce a new parameter and call it *maturing conditions*. The maturing conditions of GSNCs involved either time variations (0 h, 1.25 h, 2.5 h) at room temperature or elevated temperatures (37°C, 50°C) kept for 2.5 h. Indeed, we discovered that the *time of sample maturing* at room temperature (i.e. the delay between the sample synthesis and its purification by dialysis), possessing otherwise the same composition (i.e. reactant concentrations, their same molar ratios), being mixed exactly in the same way and under the same conditions, has a tremendous impact on their final luminescent features. When we compared our GSNCs to the identically synthesized and matured AuNCs entrapped in BSA and determined their FQY (which is more accurate than the direct comparison of fluorescence emission intensity, especially when the fluorescence maximum shifts and the width of fluorescence band changes), it turned out that the Au–Ag synergism can be achieved in specific cases exclusively.

Furthermore, inspired by the biological origin and function of the used protein, we have also investigated the influence of *physiological* (37°C) versus *elevated temperature* (50°C) in the direct comparison to room temperature maturing in the process of prolonged sample *maturing* (2.5 h). While GSNCs matured at 37°C for 2.5 h provided the best Au–Ag synergism, elevated temperature during sample maturing (50°C) led rather to changes of the protein secondary structure (determined by circular dichroism) and virtually no Au–Ag synergistic effect was observed. The results reveal that Au–Ag synergism can be finely tuned by setting appropriate maturing conditions of bimetallic nanoclusters embedded in the protein scaffold. Maturing conditions thus represent a new factor in the viewpoint of Au–Ag synergism.

For the most prominent GSNCs, revealing the best synergism, it is demonstrated that the fluorescence of GSNCs can be quenched more efficiently by an excess of Cu(II) rather than Fe(III) ions. The exact mechanism is currently unknown, but a plausible reason is suggested by us and further investigations are envisaged. Since both selected metal ions are crucial in various cellular metabolic pathways and their impaired metabolism related to several neurodegenerative diseases and/or cancer [27], their impact on GSNC represents an important issue.

Therefore, the significance and novelty of this contribution lie in unravelling a new factor, *conditions of sample maturing* (so far totally overlooked). It largely influences the Au–Ag synergism. Besides steady-state fluorescence, many other experimental techniques have been used to characterize our samples, such as inductively coupled plasma mass spectrometry (ICP-MS), X-ray photoelectron spectroscopy (XPS), UV–visible absorption spectroscopy, circular dichroism (CD), scanning transmission electron microscopy (STEM), energy-dispersive X-ray spectroscopy (EDS), dynamic light scattering (DLS) and zeta potential measurements.

## 2. Results and discussion

### 2.1. Definitions of processes called synthesis and maturing; sample labelling

**Figure 1.**
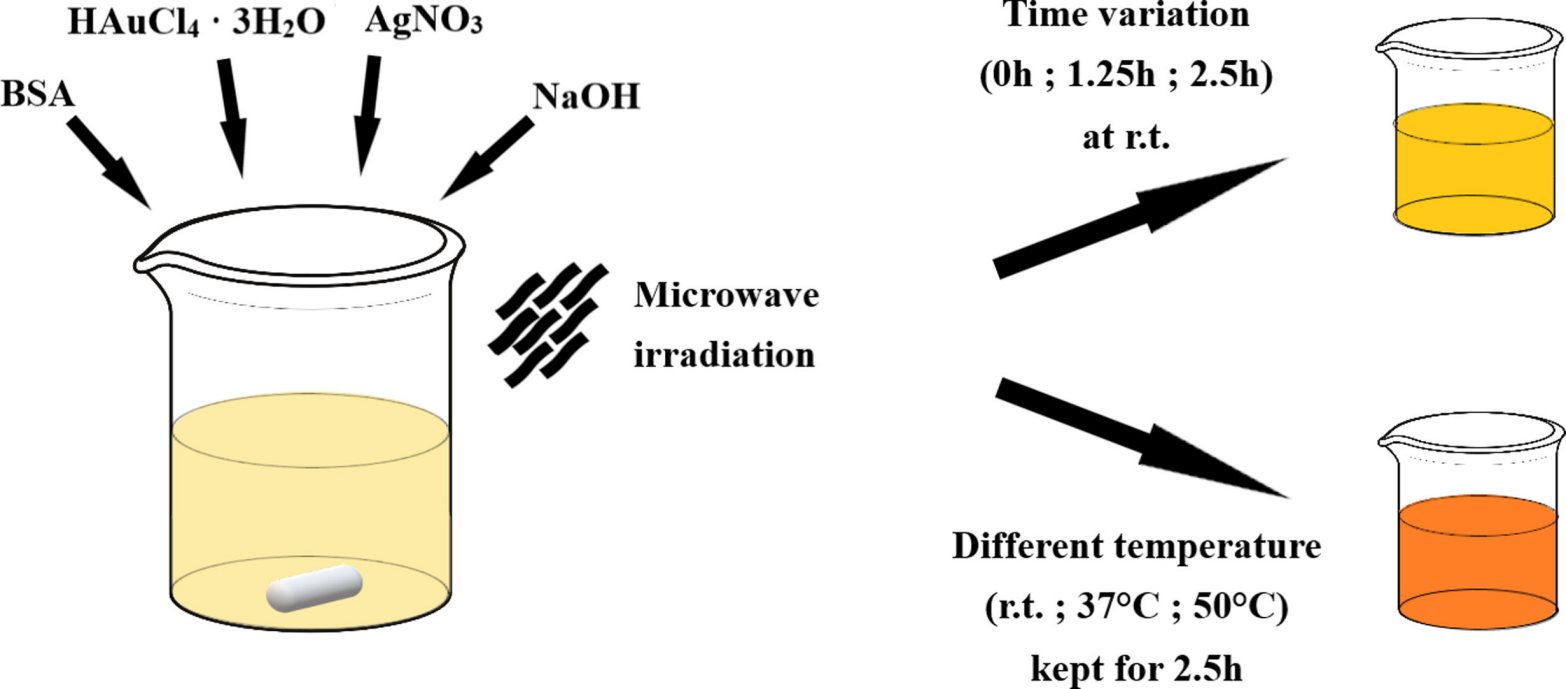
Schematic depiction of GSNC synthetic approach and then different conditions of sample maturing.

To improve luminescent properties of our previously prepared AuNCs entrapped in a protein matrix [25,26], we started to synthesize GSNC samples (figure 1) in a similar way as in [26] with two important changes: AgNO_3_ is added (to create bimetallic GSNCs) and the conditions of sample maturing, a newly introduced factor, are investigated.

Indeed, AgNO_3_ was added to a mixture of HAuCl_4_ and BSA; then, the reaction mixture was alkalized, and subjected to microwave (MW) irradiation. The metallic ion molar ratio was set to 5 : 1 (Au : Ag) based on the results of other authors reporting fluorescence emission increase due to synergism in this particular ratio (e.g. [6]). The low content of Ag within GSNCs is also intentionally kept with respect to the potential GSNC application in bio-imaging, where higher amounts of Ag ions could represent toxicity risks. Low-power MW irradiation for a short time was used to accelerate the reduction (due to tyrosine residues) of metallic ions to their oxidation state zero (and/or to uncommon oxidation states—e.g. Au(II) as evidenced by us recently [26]) under alkaline pH, and to increase NC growth. To this point, it can be called GSNC *synthesis*. Subsequently, *maturing* of the samples was allowed at room temperature (21 ± 1°C) prior to starting dialysis after 0, 1.25 or 2.5 h; the adequate samples are denoted as *GSNC 0 h RT*, *GSNC 1.25 h RT* and *GSNC 2.5 h RT*, respectively. It is envisaged that the prolonged time of sample maturing can lead to increased incorporation of metallic ions into the protein scaffold. On the other hand, too long a time of sample maturing (i.e. exceeding 2.5 h in this particular case) would lead to the overgrowing of nanoclusters into non-luminescent nanoparticles similarly as we have investigated for the case of AuNCs in our previous work [25]. In fact, one can assume that there is a limited number of binding sites for auric and silver atoms in the protein structure. Therefore, any further prolongation of sample maturing and/or increase of Au/Ag ion concentrations leads to AuNC/GSNC size increase rather than to the formation of a larger number of nanoclusters within the given protein concentration.

Based on the Arrhenius equation, any chemical reaction can be speeded up at elevated temperatures. Therefore, considering protein presence within GSNC samples, namely its natural structure and function at physiological temperature (37°C) while its tendency to denaturation and gelation at elevated temperature, the GSNC samples were also matured at 37 and/or 50°C for 2.5 h. These samples are then labelled as *GSNC 2.5 h 37°C* and *GSNC 2.5 h 50°C*, respectively. It can be assumed that elevated temperature may have an impact on the protein secondary and tertiary structures. Moreover, the elevated temperature may influence the entrapment of metallic ions to some extent because of an increased Brownian motion of ions within the sample solution.

Similarly, AuNCs in BSA were prepared and matured in the same way as GSNCs to enable a direct comparison of their characteristic properties. The monometallic samples are then labelled as *AuNCs 0 h RT*, *AuNCs 1.25 h RT* and *AuNCs 2.5 h RT*, when time of maturing varied; whereas *AuNCs 2.5 h 37°C* and *AuNCs 2.5 h 50°C* when temperature during the maturing process increased. BSA (without any metallic ions, hence non-metallic) treated by acidic/alkaline environments, MW irradiated and matured in the same way served as reference; the samples are then denoted as follows: *BSA-T 0 h RT*, *BSA-T 2.5 h RT* and *BSA-T 2.5 h 50°C*. The maturing of the samples is terminated by dialysis. After the dialysis, the purified samples are characterized by UV–visible and fluorescence spectroscopy, DLS, zeta potential measurements, ICP-MS, XPS and in selected cases by STEM equipped with EDS.

### 2.2. Characterization of the samples and evaluation of Au–Ag synergism

In the following text, we show and discuss the results of our sample characterization regarding first the inorganic (metallic) and then organic parts (represented by the protein), followed by the investigation and assessment of luminescent properties (steady-state fluorescence emission and excitation spectra, FQY determination). Finally, the estimation of particle size distribution (PDS) based on STEM imaging (dried form of the samples; only metallic parts visualized) and DLS measurements (samples in aqueous solution; the whole nanocomposite size including protein molecule and its agglomeration) will be compared and discussed.

#### 2.2.1. Metallic part characterization (by ICP-MS, XPS, UV–visible absorption)

Theoretical and experimental concentrations (in mg ml^−1^) of Au and Ag in each metallic sample (GSNC and AuNC types) are summarized in electronic supplementary material, table SI-1; while the molar ratio of Au : Ag (theoretical and experimental) for bimetallic GSNC samples is calculated in electronic supplementary material, table SI-2. It should be noted that the experimental values of Au and Ag concentrations were accurately determined by ICP-MS. Interestingly, the experimental values of Au contents slightly increased from 368 ± 4 to 381 ± 6 µg ml^−1^ with prolonged time of maturing applied at room temperature (electronic supplementary material, table SI-1). It thus corroborated the assumption that prolonged time may have an effect on metallic ion incorporation into the protein scaffold. Obviously, it is valid only for Au(III), while not for Ag(I) because the experimental content of Ag is around 40 ± 3 µg ml^−1^ in all types of GSNC samples (electronic supplementary material, table SI-1). Differences in Ag incorporation between individual GSNC samples are only negligible; they lie within the experimental error. The GSNC samples matured at elevated temperature (50°C) for prolonged time (2.5 h) did not cause any significant increase of Au content in comparison to the samples matured at room temperature for the same period (electronic supplementary material, table SI-1). On the other hand, usage of the physiological temperature of maturing led to Au(III) content slight decreasing (but within 8%). Based on the experimental concentration values of both metals, Au and Ag, the percentage yield of each metal within a particular metallic (GSNCs as well as AuNCs) sample can be calculated, the values being listed in table 1.

**Table 1.** Ratios of experimental versus theoretical (*c*(exp)/(*c*(th)) concentration values of Au and/or Ag multiplied by 100 to get the percentage yield of each metal within a particular sample.

sample	Au	Ag
	*c*(exp)/*c*(th) × 100 (%)	*c*(exp)/*c*(th) × 100 (%)
**GSNC 0 h RT**	**77.0 ± 0.8**	**76.0 ± 3.1**
**GSNC 1.25 h RT**	**70.4 ± 5.7**	**71.5 ± 5.1**
**GSNC 2.5 h RT**	**79.0 ± 1.2**	**79.5 ± 1.4**
**GSNC 2.5 h 37°C**	**73.8 ± 1.2**	**76.3 ± 2.0**
**GSNC 2.5 h 50°C**	**80.6 ± 0.2**	**76.9 ± 6.0**
*AuNCs 0 h RT*	*77.5 ± 1.5*	—
*AuNCs 1.25 h RT*	*75.4 ± 1.5*	—
*AuNCs 2.5 h RT*	*79.2 ± 2.8*	—
*AuNCs 2.5 h 37°C*	*73.4 ± 1.2*	—
*AuNCs 2.5 h 50°C*	*79.5 ± 2.9*	—

Table 1 clearly shows that gold incorporation into the resulting bimetallic nanocomposites lies between 70.4 and 80.6% (experimental errors 5.7%). In comparison to that, the percentage of gold incorporation in monometallic samples is narrower, within 73.4–79.5% (experimental error 2.9%). Since both intervals mutually overlap, it can be stated that gold incorporation is not influenced by silver addition into the reaction mixture. Ag is incorporated by 71.5 (±5.1)–79.5 (±1.4)% into bimetallic GSNC samples, i.e. quite constantly (within the experimental error) regardless of maturing conditions. Based on the ICP-MS results, it could be concluded that silver cation entrapment is less influenced by maturing conditions than gold ones.

**Figure 2.**
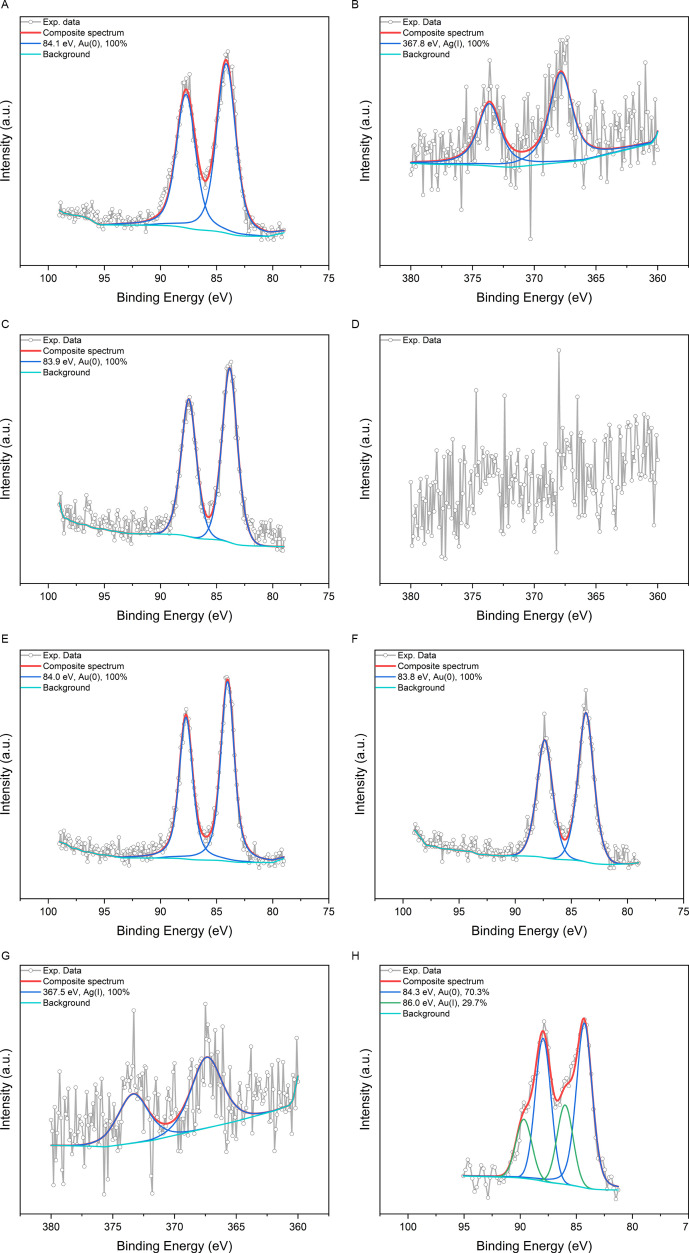
XPS signals in Au and Ag characteristic regions recorded for our representative metallic samples: (*A*) Au 4f region of GSNC 0 h RT, (*B*) Ag 3d region of GSNC 0 h RT, (*C*) Au 4f region of GSNC 1.25 h RT, (*D*) Ag 3d region of GSNC 1.25 h RT, (*E*) Au 4f region of GSNC 2.5 h RT, (*F*) Au 4f region of GSNC 2.5 h 37°C, (*G*) Ag 3d region of GSNC 37°C, and (*H*) Au 4f region of GSNC 2.5 h 50°C.

To determine oxidation states of Au and Ag within our metallic samples, XPS measurement has been employed. The XPS signals of GSNC samples are shown in figure 2, while those of AuNC samples in electronic supplementary material, figure SI-1.

XPS signal fitting revealed Au(0) in all metallic samples (AuNCs as well as GSNCs) regardless of the maturing conditions with the only exception being GSNC 2.5 h 50°C, where a mixture of Au(I) and Au(0) was detected (around 70% of Au(0) and the rest for Au(I)). It is in accordance with the literature, since Au(0) has been frequently detected in bimetallic Au–Ag–BSA systems employing XPS [4,11,16]. However, it has to be recalled here that XPS may change the oxidation state of metallic atoms to some extent in BSA-protected metallic nanoclusters [26]. In our previous work [26], we used electron paramagnetic resonance (EPR) spectroscopy, a mild, but very sensitive technique in comparison to XPS, to investigate AuNCs embedded in BSA. According to the EPR spectra simulation and interpretation, Au is present not only as Au(0), but also as Au(II); the presence of Au(I) cannot be excluded either [26]. Therefore, comparing our XPS results obtained for AuNCs in this work (the synthetic procedure is basically the same as in [26]), it could be envisaged that besides Au(0), there may also be Au(I) and Au(II) present in GSNC samples to some extent, but below the detection limit of XPS. Furthermore, under the assumption that we are using the same synthetic and maturing procedures of bi-/monometallic samples of a particular type, any detectable changes in the content of Au(0), Au(I) and Au(II) can be caused solely by the presence/absence of Ag(I). This can be demonstrated on GSNC 2.5 h 50°C sample (figure 2H; around 30% of Au(I) and 70% of Au(0) detected) in comparison to XPS signal recorded from its referent sample, i.e. AuNCs 2.5 h 50°C (electronic supplementary material, figure SI-1C; 100% Au(0) detected).

Very weak signals of Ag have been detected in GSNC 0 h RT, GSNC 1.25 h RT and GSNC 2.5 h 37°C; whereas no Ag peaks were observed in the other two GSNC samples unfortunately. Based on the Ag peak position at 367.8 eV in GSNC 0 h RT sample, Ag(I) oxidation state has been assigned (figure 2B) according to the literature [28]. On the other hand, Ag(II) may be encountered in GSNC 2.5 h 37°C sample if we consider the fit at 367.4 eV as valid (very noisy XPS signal). The weak signal of Ag might be related to its low concentration (approximately 40 µg ml^−1^; see electronic supplementary material, table SI-1) within the samples, as well as to the presence of the protein. Indeed, the secondary structure of the protein changes during maturing of GSNCs for a prolonged time and/or at elevated temperatures as will be discussed later. This possibly leads to silver incorporation inside the protein and, consequently, Ag becoming invisible for XPS. Thus, the detection limit for Ag in XPS measurements is not exceeded in GSNC 2.5 h RT and GSNC 2.5 h 50°C. Complementary to XPS, X-ray absorption near-edge spectroscopy (XANES) would be a particularly useful and effective technique for probing the valence state of atoms, especially those that are difficult to differentiate by XPS, such as Ag, as stated in [6]. However, we do not have any access to XANES. Hence, from our current experimental data (based on XPS solely), it can be concluded that the presence of silver cations changes the oxidation state of Au in GSNC 2.5 h 50°C sample only. For the other samples and maturing conditions, Ag(I) does not influence the oxidation state of Au within GSNC.

**Figure 3.**
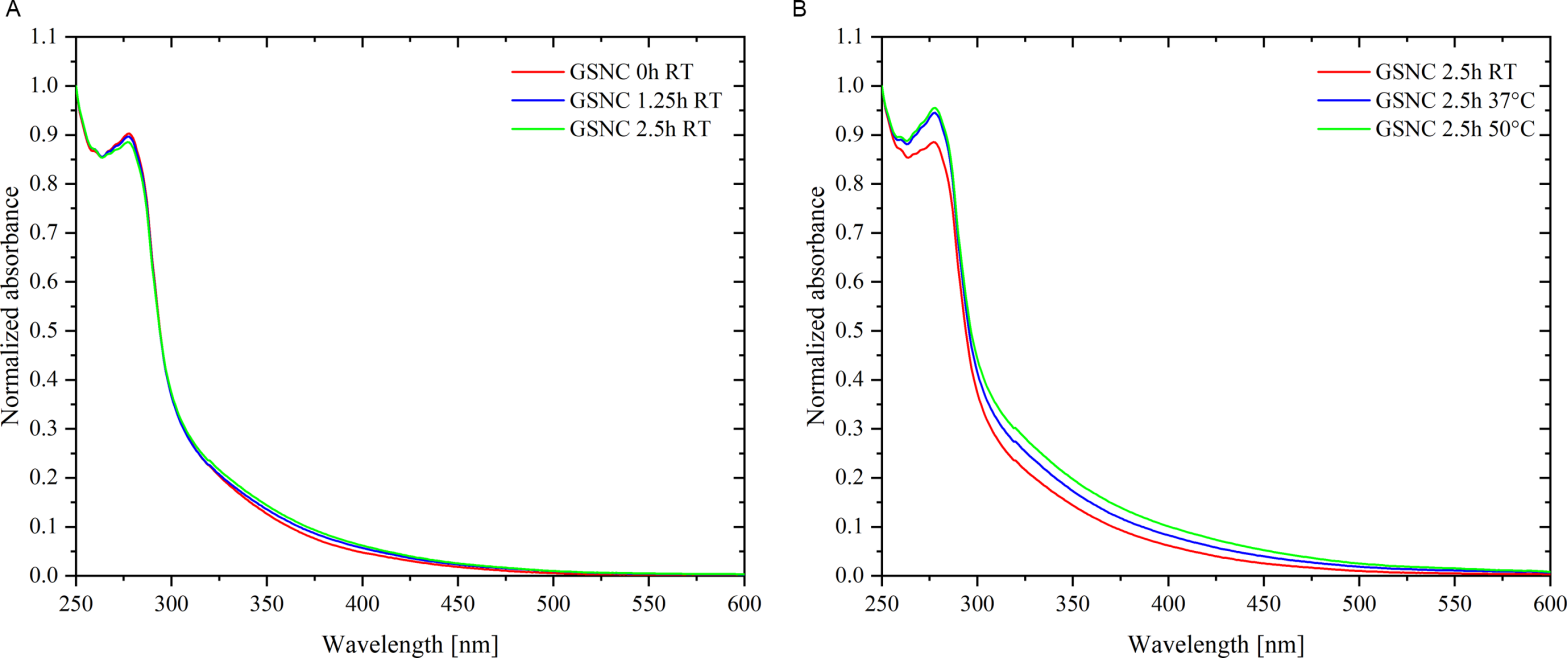
Absorption spectra of GSNC matured either (*A*) at room temperature (RT) for 0, 1.25 and 2.5 h; or (*B*) at RT versus 37°C versus 50°C for 2.5 h. Spectra were normalized with respect to the absorption at 250 nm.

With respect to the detection of zero-valent Au (determined by XPS), one should consider the potential metallic character of Au nanostructures within our GSNC and AuNC samples of different maturing conditions. Au(0) atoms assembled into nanostructures may form either nanoclusters (sizes not exceeding 1.8 nm in diameter) and/or plasmonic nanoparticles (bigger sizes than nanoclusters), possessing surface plasmon resonances (SPR) in the visible region of electromagnetic radiation (for instance, located at around 520 nm for Au particles of 10−50 nm in diameter). Figure 3 and electronic supplementary material, figure SI-2, clearly prove that SPR band is missing, i.e. nanoclusters and not nanoparticles are present in all metallic samples.

In the UV part of the absorption spectra of GSNCs presented in figure 3, absorption with the maximum located at around 280 nm, stemming from the protein, is clearly observed. Additionally, in all five GSNC samples, absorption is increased in the region of 300–500 nm creating thus a tail to the main band in the UV region. This correlates well with the observations of others (e.g. [15]) and indicates that metallic nanostructures are formed within GSNCs. Importantly, there is a substantially greater absorption increase within the 300–500 nm region in samples matured at 37°C and/or 50°C for 2.5 h (figure 3B). One of the plausible explanations may be an increase of metallic nanostructure diameter as will be discussed later. Non-metallic BSA-T samples (serving as references) were measured using a UV–visible absorption spectrometer as well and their spectra are shown for the sake of a direct comparison with those of metallic samples in electronic supplementary material, figure SI-2. Intentionally, the absorption spectra of GSNC, AuNC and BSA-T samples are assembled into one picture according to the specific maturing conditions (see electronic supplementary material, figure SI-2).

#### 2.2.2. Organic part characterization: changes of protein secondary structure

Let us now have a look at the organic part of the samples being matured under different conditions. Since the matrix for our metallic nanocluster generation is represented by the protein that is robust, but prone to structural changes upon acidifying/alkalization/heating [25], it is reasonable to investigate it using CD.

CD spectra were measured for all metallic samples and their appropriate references (BSA-T) and compared with natural BSA as shown in electronic supplementary material, figure SI-3. Using the BeStSel program (as in our previous work [25]), the percentage contents of α-helices, antiparallel/parallel structures, turns and others were evaluated. Then, table 2 clearly summarizes the percentage contents of each structural arrangement of the protein scaffold within our metallic as well as non-metallic samples.

**Table 2.** Estimation of the percentage contents of structural arrangements of protein (using the BeStSel program) in GSNCs, AuNCs and BSA-T under different maturing conditions compared with natural BSA. The estimated error of structural arrangement determination is around 1%.

sample	content (%)						
	helix 1 (regular)	helix 2 (distorted)	anti 2 (relaxed)	anti 3 (right-twisted)	parallel	turns	others
BSA	41.0	20.1	0.0	0.0	0.0	9.7	29.2
BSA-T 0 h RT	18.4	14.6	1.7	5.6	4.8	11.4	43.5
*AuNCs 0 h RT*	*17.1*	*13.4*	*4.3*	*7.0*	*4.2*	*12.4*	*41.6*
**GSNC 0 h RT**	**15.5**	**13.5**	**4.3**	**6.7**	**5.2**	**11.4**	**43.4**
BSA-T 1.25 h RT	23.5	15.0	1.7	5.1	1.0	11.0	42.7
*AuNCs 1.25 h RT*	*18.8*	*14.1*	*1.1*	*7.1*	*3.0*	*12.3*	*43.3*
**GSNC 1.25 h RT**	**15.8**	**13.0**	**3.7**	**7.9**	**4.1**	**12.2**	**43.3**
BSA-T 2.5 h RT	14.3	11.7	6.3	8.6	6.3	11.3	41.5
*AuNCs 2.5 h RT*	*14.9*	*12.4*	*4.0*	*8.5*	*5.1*	*12.4*	*42.8*
**GSNC 2.5 h RT**	**12.9**	**10.9**	**7.3**	**9.5**	**6.4**	**11.8**	**41.8**
BSA-T 2.5 h 37°C	15.2	12.1	4.8	8.1	4.1	12.3	43.4
*AuNCs 2.5 h 37°C*	*13.4*	*11.6*	*2.8*	*10.7*	*4.5*	*14.2*	*42.7*
**GSNC 2.5 h 37°C**	**12.0**	**11.1**	**4.6**	**10.1**	**4.9**	**12.6**	**44.6**
BSA-T 2.5 h 50°C	9.7	8.4	7.7	12.3	5.0	13.0	43.9
*AuNCs 2.5 h 50°C*	*9.7*	*7.2*	*6.2*	*12.8*	*5.1*	*13.5*	*45.5*
**GSNC 2.5 h 50°C**	**8.5**	**6.1**	**9.3**	**14.0**	**3.7**	**14.0**	**44.5**

The secondary structure of BSA changes substantially as seen in table 2. Indeed, α-helicity reduces significantly when treating BSA by conditions of synthesis and maturing of metallic samples, i.e. compare BSA-T samples with BSA (in table 2). Simultaneously, the other structures (the last column in table 2) increase their percentage contents adequately. The most pronounced decrease of α-helicity and simultaneous increase of less-ordered structures is observed in BSA-T 2.5 h 50°C. This means that synthetic conditions and maturing under elevated temperature for prolonged time influence the secondary structure of the protein to the largest extent.

By introducing gold atoms into BSA (rows in italic in table 2), further changes of protein secondary structure toward a less-ordered one occur (compare BSA-T and AuNC samples, rows in italic in table 2, matured under the same conditions, e.g. 0 h RT etc.). The changes are, however, less dramatic than those observed between natural BSA and BSA-T (table 2) discussed in the previous paragraph. This perfectly correlates with our observation made for AuNCs measured by CD right after the synthesis (i.e. without any maturing step) [25]. The most pronounced changes are again observed for AuNCs 2.5 h 50°C (when compared with AuNCs 0 h RT). Consistent with the above-discussed BSA-T samples, the secondary structure of the protein in AuNC samples is significantly altered when maturing at 50°C used for prolonged time.

Similar characteristic trends as in AuNCs are observed when CD spectra of GSNC samples (rows in bold in table 2) are recorded and compared with BSA-T samples (table 2): reduction of α-helicity with simultaneous increase of the other structure contents, namely right-twisted anti parallel arrangement.

Looking into the scientific literature, 16.5% of regular α-helix in AuAg–BSA system, prepared by stirring for 4−8 h at room temperature, was reported [16]. This value is in full accordance (considering that the experimental error is around 1%) with our data for GSNC 1.25 h RT (indeed, NC formation and concomitant BSA structural changes are faster due to MW irradiation employed in our sample preparation procedure): 15.8% of regular α-helix (table 2). Evidence of substantial changes of protein secondary structure when AuAg–BSA nanocomposites formed was given by Dutta *et al*. as well [15]. Therefore, there is a reasonable correlation of our data with that in the scientific literature dealing with AuAg–BSA nanocomposites.

#### 2.2.3. Luminescent properties of gold–silver nanoclusters and extent of Au–Ag synergism

**Figure 4.**
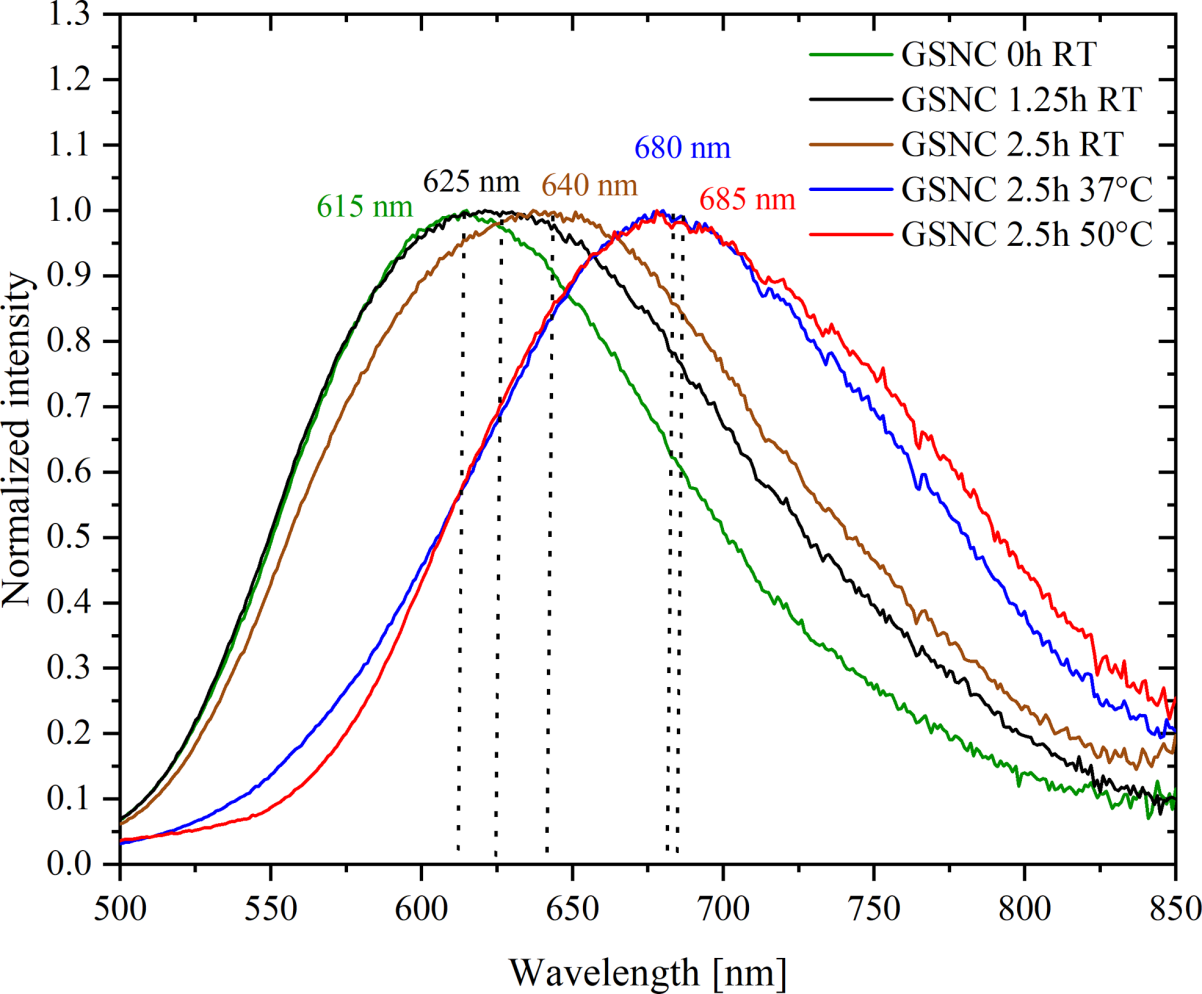
Fluorescence emission of GSNC samples matured under different conditions: 0 h RT (green curve), 1.25 h RT (black curve), 2.5 h RT (brown curve), 2.5 h 37°C (blue curve) and 2.5 h 50°C (red curve). Excitation was at 460 nm in all five cases. Normalized spectra are shown for the sake of a clear presentation of intensity maximum shifts and emission band broadening.

Fluorescence emission was measured using the same excitation wavelength (460 nm) to investigate luminescent properties of GSNC samples generated under different maturing conditions. Normalized spectra are depicted in figure 4.

**Figure 5.**
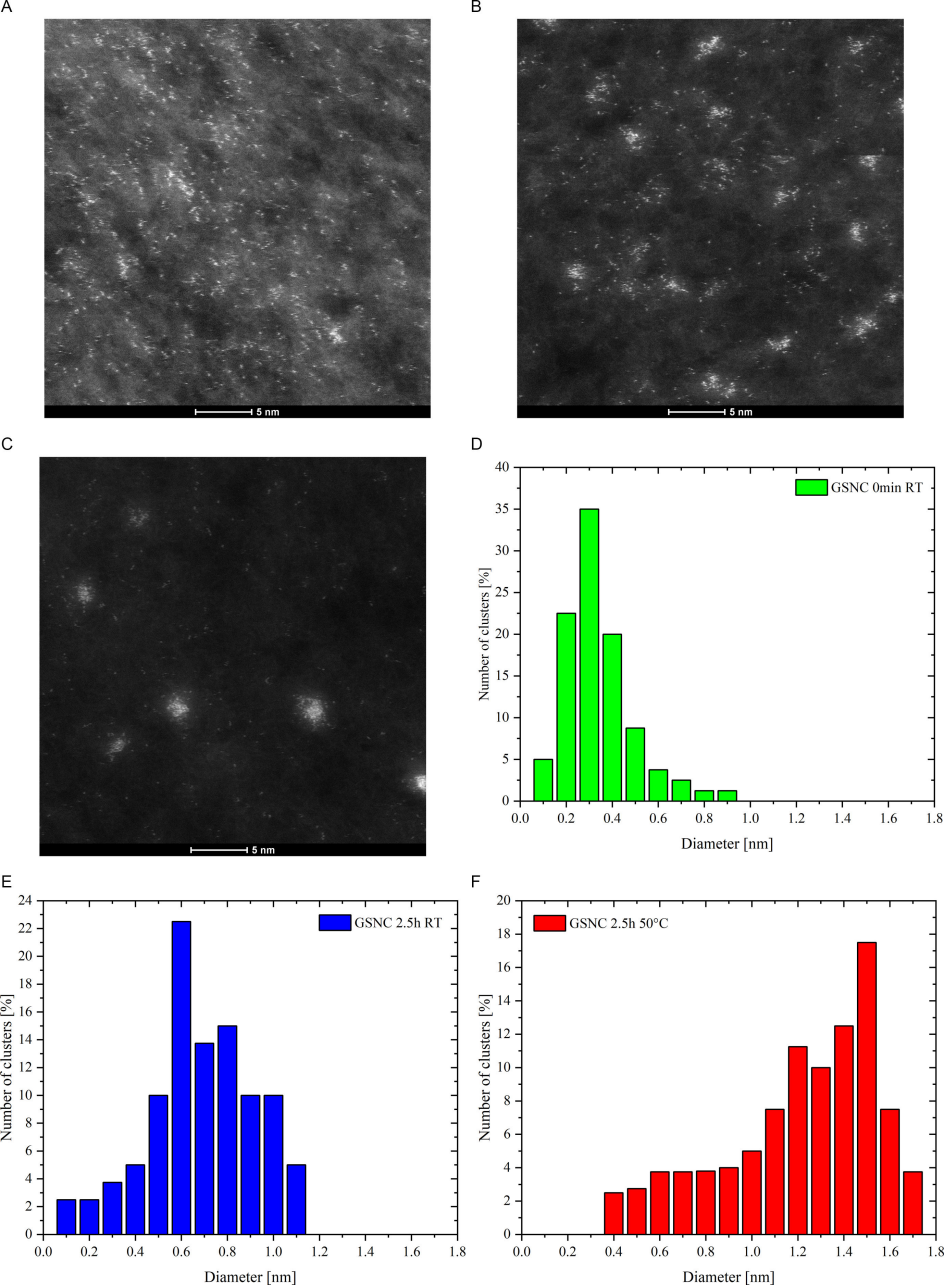
STEM images of GSNC samples matured under different conditions: (*A*) 0 h RT, (*B*) 2.5 h RT and (*C*) 2.5 h 50°C. PSD diagrams derived from STEM images: (*D*) from (*A*) image, (*E*) from (*B*) image and (*F*) from (*C*) image.

The position of emission maximum of GSNC 0 h RT sample is located at 615 nm (figure 4). This corresponds very well to the position of fluorescence emission maxima reported for Au : Ag systems (also 5 : 1 ratio) prepared by Zhang *et al*., albeit they exploited peptide (GSH) as the template for nanocluster synthesis [6]. While prolonging the maturing time, the emission maximum red-shifts to 625 nm and further to 640 nm (figure 4) as obvious for GSNC 1.25 h RT and GSNC 2.5 h RT, respectively. While increasing the maturing temperature, the emission maximum continues to red-shift to 685 nm (GSNC 2.5 h 50°C). Similarly, the emission maxima of our monometallic AuNC samples red-shifted gradually either by 5 nm (going from samples matured at room temperature for 0 h (655 nm) to that matured for 2.5 h at 37°C (670 nm)), or by 10 nm (for the AuNCs matured at 50°C for 2.5 h) as can be clearly seen in electronic supplementary material, figure SI-4. Based on the well-known theory for AuNCs [29], the position of emission maxima can be related to the size of luminescent nanoclusters: the bigger the nanoclusters, the greater the red-shift of their emission maximum. Therefore, it can be derived from fluorescence measurements that luminescent nanoclusters within GSNC samples grow into bigger ones under maturing conditions in the following order: 0 h RT < 1.25 h RT < 2.5 h RT < 2.5 h 37°C < 2.5 h 50°C. This was confirmed by STEM imaging shown in figure 5A*–*C for the selected samples (i.e. the samples placed at margins and in the centre of this derived order).

The augmentation of luminescent nanocluster sizes is also indirectly evidenced by measuring excitation spectra of all metallic samples—see electronic supplementary material, figure SI-5. In fact, the excitation spectrum measured for a specific emission wavelength of a particular sample provides us with information about the contribution of the selected emissive species to the overall absorption of the sample observed in figure 3. It is like picking up only one species and distinguishing its absorption properties in a mixture without the necessity of its physical separation. It should be emphasized here that the sensitivity of the spectrofluorometer is higher than that of the UV–visible absorption spectrophotometer. This means that tiny differences in GSNC and/or AuNC sizes and their PSD will be more easily detected and reflected in excitation rather than in absorption spectra (compare figure 3 with electronic supplementary material, figure SI-5). Hence, there is an obvious red-shift of the band positioned at around 500 nm in AuNC cases when going from sample labelled 0 h RT to 2.5 h 50°C (electronic supplementary material, figure SI-5B), reporting about an increasing size of luminescent nanoclusters. Evidently, this red-shift is masked to a large extent in the case of GSNC samples (electronic supplementary material, figure SI-5A). We assume that this is due to the presence of silver in GSNC samples. The bands within the excitation spectra of GSNC, namely in the visible region, are less resolved, although the trend indicating nanocluster size increase and PSD broadening, when maturing sample at elevated temperature for prolonged time, still holds. Importantly, there is a slight blue-shift of the UV band positioned at approximately 305 nm observed only in GSNC 2.5 h 50°C in electronic supplementary material, figure SI-5A. It might be related to the presence of Ag and concomitant conditions of sample maturing (50°C for 2.5 h) because it is not observed in AuNCs 2.5 h 50°C (electronic supplementary material, figure SI-5B). The origin of this UV band might be ascribed to luminescent AuAg organometallic complexes and/or to very tiny nanoclusters (literally, 2−3 metallic atoms interacting mutually).

Moreover, the plot of normalized fluorescence emission intensity (figure 4) gives direct evidence that a broader PSD of luminescent nanoclusters occurs within GSNC samples matured for prolonged time (2.5 h), because bigger full-width-at-half-maximum (FWHM) values can be derived for GSNC 2.5 h RT (FWHM = 186 nm; electronic supplementary material, table SI-3) than that for GSNC 0 h RT (FWHM = 151 nm; electronic supplementary material, table SI-3). It is not surprising because prolonged time of GSNC sample maturation means prolonged access of metallic ions (Au(III) and Ag(I)) to the protein residues, resulting thus in PSD broadening and size increase of luminescent nanoclusters. This is corroborated by STEM imaging and consequent PSD evaluation (depicted in figure 5D*–*F) as will be discussed later.

To quantify synergism of Au–Ag in GSNC samples objectively (i.e. excluding any influence of fluorescent experimental setup and other technical issues), we determined FQY of mono- and bimetallic samples; they are listed in table 3. For the sake of a clear comparison and evaluation of Au–Ag synergism, we expressed the ratio of FQY values obtained for GSNC samples divided by that of AuNCs (table 3). Obviously, Au–Ag synergism is the highest in the sample matured for 2.5 h at 37°C; while the lowest is in GSNC 0 h RT. Therefore, it can be concluded that 2.5 h maturing time of the samples kept at physiological temperature is very convenient for the observation of Au–Ag synergism. On the contrary, the augmented temperature of sample maturing (50°C) and/or too short maturing times (0 h, 1.25 h) are unfavourable for the Au–Ag synergism (table 3). The former may be related to the substantial changes of the protein secondary structure (not only in metallic, but also in the referent system) induced by the conditions of synthesis and maturing (performed at elevated temperature far exceeding the physiological one) as evidenced in table 2. The latter may be caused by the slow formation of nanoclusters and their PSD broadening (as derived from UV-visible and luminescence spectra). From the above-shown and discussed results it can be deduced that rather than the real Ag content and its ratio to the real Au content (determined by ICP-MS), a precise inner structure of Au–Ag nanoclusters embedded in the protein matrix influences the so-called synergism of Au and Ag. Thus, to sum up, the inner structure of Au–Ag nanocomposites can be finely tuned by maturing conditions.

**Table 3.** Fluorescence quantum yield (FQY) of bi- and monometallic samples and changes in Au–Ag synergism expressed as FQY(GSNCs) : FQY(AuNCs) ratio.

sample	FQY (%)	FQY(GSNCs)/FQY(AuNCs)
**GSNC 0 h RT**	**5.6**	**0.69**
**GSNC 1.25 RT**	**6.0**	**1.03**
**GSNC 2.5 h RT**	**4.1**	**1.78**
**GSNC 2.5 h 37°C**	**5.6**	**2.07**
**GSNC 2.5 h 50°C**	**2.7**	**1.08**
*AuNCs 0 h RT*	*8.1*	—
*AuNCs 1.25 RT*	*5.8*	—
*AuNCs 2.5 h RT*	*2.3*	—
*AuNCs 2.5 h 37°C*	*2.7*	—
*AuNCs 2.5 h 50°C*	*2.5*	—

#### 2.2.4. Particle size distribution changes due to maturing conditions and zeta potential values

Based on our experience with high-resolution transmission electron microscopy (HR-TEM) imaging of AuNCs [26], we visualized GSNC samples rather by STEM so that the samples are not modified by the focused electron beam. The representative STEM images are shown in figure 5A*–*C. Moreover, EDS was measured at several places within several HAADF maps and revealed the presence of Au as well as Ag in GSNC samples (electronic supplementary material, figure SI-6). The other elements stem from either microscopic copper grid covered by holey carbon, or from the samples.

Evidently, GSNC sample matured at room temperature for prolonged time (figure 5B) in comparison to GSNC 0 h RT (figure 5A) contains more agglomerates of bright spots (representing Au/Ag atoms in protein matrix deposited on holey carbon-covered copper grid for STEM imaging). The same trend and more dense agglomerates of bright spots are observed in GSNC sample matured at augmented temperature (figure 5C) when compared with GSNC 2.5 h RT (figure 5B). Therefore, the increased sizes of luminescent nanostructures within GSNC samples derived from fluorescence and UV–visible absorption measurements are well evidenced and visualized by STEM. Considering the very slightly increasing Au content and virtually constant Ag content as determined by ICP-MS (electronic supplementary material, table SI-1), it can be assumed that bigger particles (i.e. more dense agglomerates of bright spots) appeared at the expense of smaller ones.

Furthermore, PSD of metallic agglomerates was determined based on STEM imaging. The resulting histograms are shown in figure 5D*–*F for GSNC 0 h RT, GSNC 2.5 h RT and GSNC 2.5 h 50°C, respectively. The histograms give direct proof about the size increase of metallic nanostructure entities and PSD broadening when going from GSNC 0 h RT, through GSNC 2.5 h RT, to GSNC 2.5 h 50°C. The narrowest PSD of metallic agglomerates was observed in GSNC 0 h RT sample as has been already envisaged from the discussion of luminescent properties. Thus, the microscopic data well correlate mutually and confirm the changes within GSNC samples derived from luminescent measurements and induced by the specified maturing conditions.

If one considers the average length of Au–Au and/or Au–Ag bonds (0.282 and/or 0.278 nm, respectively) determined by X-ray absorption spectroscopic (XAS) methods [30,31], the bright spots of approximately 0.14 nm in diameter (frequently encountered in GSNC 0 h RT) should represent one noble metal atom in STEM images shown in figure 5. Then, the term organometallic nanocomplexes rather than nanoclusters should be used for such tiny objects. Moreover, there is a vast literature concerning Au(I) complexes that possess luminescent properties due to aurophilic interaction [32]. Therefore, further investigations are necessary to distinguish between luminescent organometallic complexes and luminescent nanoclusters; indeed, how to define the border between these two luminescent entities.

PSD of all metallic (GSNC and AuNC) and nonmetallic (BSA-T) samples was also determined using DLS, measured directly in the diluted aqueous solutions. Average values of particle sizes in BSA-T, AuNC (rows in italic), and GSNC (rows in bold) samples recorded using DLS as number and intensity changes are clearly summarized in table 4. It should be recalled that the hydrodynamic diameter of the whole nanocomposite (protein + luminescent nanoclusters +o rganometallic complexes + hydration envelope of the nanocomposite) is estimated via DLS measurements, while contrast of metallic elements (only) is visualized in STEM. Consequently, the exact values of nanostructure diameters determined by STEM versus by DLS differ and PSD can be shifted significantly.

**Table 4.** Average values of particle sizes of metallic and non-metallic samples determined by DLS and processed according to number and intensity changes (percentage contents are given in parentheses). Zeta potential values are listed as well.

sample	size (number) (nm)	size (intensity) (nm)				zeta potential (mV)
BSA-T 0 h RT	1.7 ± 0.1	636.2 ± 16.1 (16.1%)	22.6 ± 0.7 (41.1%)	—	2.4 ± 0.1 (42.8%)	ND
*AuNCs* *0 h RT*	*1.6 ± 0.1*	*527.7 ± 45.0* *(54.3%)*	*57.8 ± 4.5* *(30.4%)*	*15.0 ± 2.6* *(6.6%)*	*2.0 ± 0.1* *(8.7%)*	*−40.5 ± 1.0*
**GSNC** **0 h RT**	**2.6 ± 0.3**	**512.3 ± 19.1** **(36.1%)**	**47.4 ± 4.1** **(53.3%)**	—	**3.4 ± 0.4** **(10.6%)**	**−26.9 ± 0.6**
BSA-T 1.25 h RT	1.2 ± 0.1	134.2 ± 23.4 (9.8%)	26.2 ± 1.0 (50.8%)	—	1.7 ± 0.1 (39.4%)	−29.3 ± 2.9
*AuNCs 1.25 h RT*	*1.5 ± 0.1*	*473.8 ± 41.9* *(52.4%)*	*54.0 ± 4.4* *(28.6%)*	*16.0 ± 2.1* *(8.7%)*	*1.8 ± 0.1* *(10.3%)*	*−40.4 ± 1.0*
**GSNC 1.25 h RT**	**1.4 ± 0.2**	**437.0 ± 70.9** **(44.9%)**	**52.3 ± 2.0** **(37.3%)**	**12.0 ± 1.2** **(6.6%)**	**1.8 ± 0.2** **(11.2%)**	**−40.0 ± 1.2**
BSA-T 2.5 h RT	1.7 ± 0.2	457.8 ± 17.0 (75.6%)	21.0 ± 1.1 (14.0%)	—	2.3 ± 0.2 (10.4%)	−34.9 ± 0.1
*AuNCs 2.5 h RT*	*1.6 ± 0.1*	*522.0 ± 19.4* *(79.1%)*	*38.6 ± 2.7* *(14.6%)*	—	*1.7 ± 0.1* *(6.3%)*	*−39.6 ± 0.4*
**GSNC 2.5 h RT**	**1.9 ± 0.3**	**487.9 ± 34.9** **(68.8%)**	**37.3 ± 1.8** **(24.3%)**	—	**2.3 ± 0.4** **(6.9%)**	**−34.3 ± 1.1**
BSA-T 2.5 h 37°C	1.3 ± 0.1	549 ± 22.4 (77.9%)	26.9 ± 2.4 (13.0%)	—	1.5 ± 0.1 (9.1%)	−42.7 ± 0.4
*AuNCs 2.5 h 37°C*	*1.5 ± 0.2*	*385.8 ± 26.1* *(64.4%)*	*41.8 ± 4.2* *(20.5%)*	*13.7 ± 2.9* *(5.7%)*	*1.9 ± 0.2* *(9.4%)*	*−40.5 ± 0.6*
**GSNC 2.5 h 37°C**	**1.3 ± 0.2**	**457.2 ± 25.3** **(75.6%)**	**40.5 ± 2.5** **(18.1%)**	—	**1.6 ± 0.2** **(6.3%)**	**−41.3 ± 0.4**
BSA-T 2.5 h 50°C	20.9 ± 4.4	503.6 ± 34.0 (92.5%)	25.5 ± 5.0 (7.5%)	—	—	−38.0 ± 0.6
*AuNCs 2.5 h 50°C*	*25.1 ± 3.2*	*460.6 ± 16.8* *(90.8%)*	*31.2 ± 4.5* *(9.2%)*	—	—	*−37.9 ± 0.5*
**GSNC 2.5 h 50°C**	**23.1 ± 6.3**	**413.0 ± 13.0** **(86.7%)**	**34.6 ± 3.0** **(13.3%)**	—	—	**−38.6 ± 0.8**

DLS involves auto-correlating the intensity changes of the scattered light as a function of time, and thus the solely measured PSD values are based on intensity, while the others are derived from it, by taking several assumptions into account, such as spherical shape of particles within the samples for instance. Nevertheless, it is a good approximation in this case. Let us discuss changes in PSD based on number first. As for the referent samples (non-metallic, BSA-T, in table 4), PSD does not change much due to the prolonged maturing of the samples at room and/or physiological temperatures, i.e. it reveals values of a few nanometres. On the contrary, PSD based on number sharply increased to 20.9 ± 4.4 nm in BSA-T 2.5 h 50°C. A very similar trend of PSD changes was observed in AuNC (rows in italic in table 4) and GSNC (rows in bold in table 4) samples: even more pronounced abrupt PSD increase in samples matured at 50°C for 2.5 h (to 25.1 ± 3.2 nm and to 23.1 ± 6.3 nm, respectively).

As is evident from table 4, PSD based on intensity changes measured by DLS reveals mostly a trimodal character for non-, mono- and bi-metallic samples matured at room temperature (with the exceptions represented by AuNCs 0 h RT, AuNCs 1.25 h RT, GSNC 1.25 h RT, where even four different sizes were detected): sizes of particles reaching a few, dozens and hundreds of nanometres are observed. This can be even visualized graphically as shown in electronic supplementary material, figure SI-7. Considering the trends of percentage contents of a particular particle size (listed in parentheses in table 4) in conjunction with the specified maturing conditions, one can see that the percentage content of sizes reaching hundreds of nanometres increases with prolonged time of maturing in metallic samples as well as referent systems.

On the contrary, almost bimodal instead of trimodal character of PSD is encountered in samples matured at 37°C and 50°C: sizes of particles of dozens and hundreds of nanometres remained, while those of a few nanometres disappeared in comparison to the samples matured at room temperature (table 4). The overall increase of particle sizes is also evident from the augmented percentage contents of particles manifesting themselves by sizes of several hundreds of nanometres in samples matured at 50°C. It can be deduced that the increasing average size within PSD correlates with the changes of maturing conditions that induce conformational changes of the protein scaffold.

Comparing our DLS results obtained for GSNCs with those published in scientific literature, an average hydrodynamic diameter of 3.2 nm (determined by DLS) was reported for GSH–AuAg nanocomposites in [6]. AuAg nanostructures of average sizes around 4 nm in diameter (over 90%) based on intensity changes were reported in [3]. Hence, our nanocomposites are bigger than the reported values which may be caused by the presence of the protein in our work in comparison to the literature [6] where the tripeptide is used; while by the procedures of synthesis (using MW) and maturing (the significance and novelty of this work) in comparison to the other literature [3].

It can be summarized, based on DLS measurements, that the maturing conditions have a great impact on PSD. The trend of the increasing average particle sizes that can be derived from DLS corresponds very well with the results of STEM imaging as well as with the changes of luminescent properties (namely, the position of emission maximum) of mono- and bi-metallic samples shown and discussed above.

Last, but not least, zeta potential values of our non-, mono- and bi-metallic samples were measured, the resulting average values being listed in the last column of table 4. Importantly, zeta potentials fall below −26 mV in all measured samples regardless the composition and maturing conditions (table 4). This means, in turn, that in all measured samples, nanostructures are well stabilized electrostatically in aqueous solutions. It agrees with the observation made by the naked eyes: no precipitation or turbidity was observed even if the samples were stored for several months (in a fridge due to the presence of the protein). Negative zeta potential value (around −28 mV) of luminescent AuAg nanocomposites has been also reported in the scientific literature [6]. As for the potential impact of maturing conditions on zeta potential values, there was not any distinguishable simple trend that would be repeatable for metallic nanoclusters, since the values oscillated in the interval from −26.9 to −41.3 mV in GSNC samples; while from −40.5 to −37.9 mV in AuNC samples (table 4). Due to the complexity of the prepared nanocomposites, it is difficult to find any reasoning for the zeta potential value fluctuations.

### 2.3. Luminescence quenching of gold–silver nanoclusters induced by Cu(II) and/or Fe(III)

**Figure 6.**
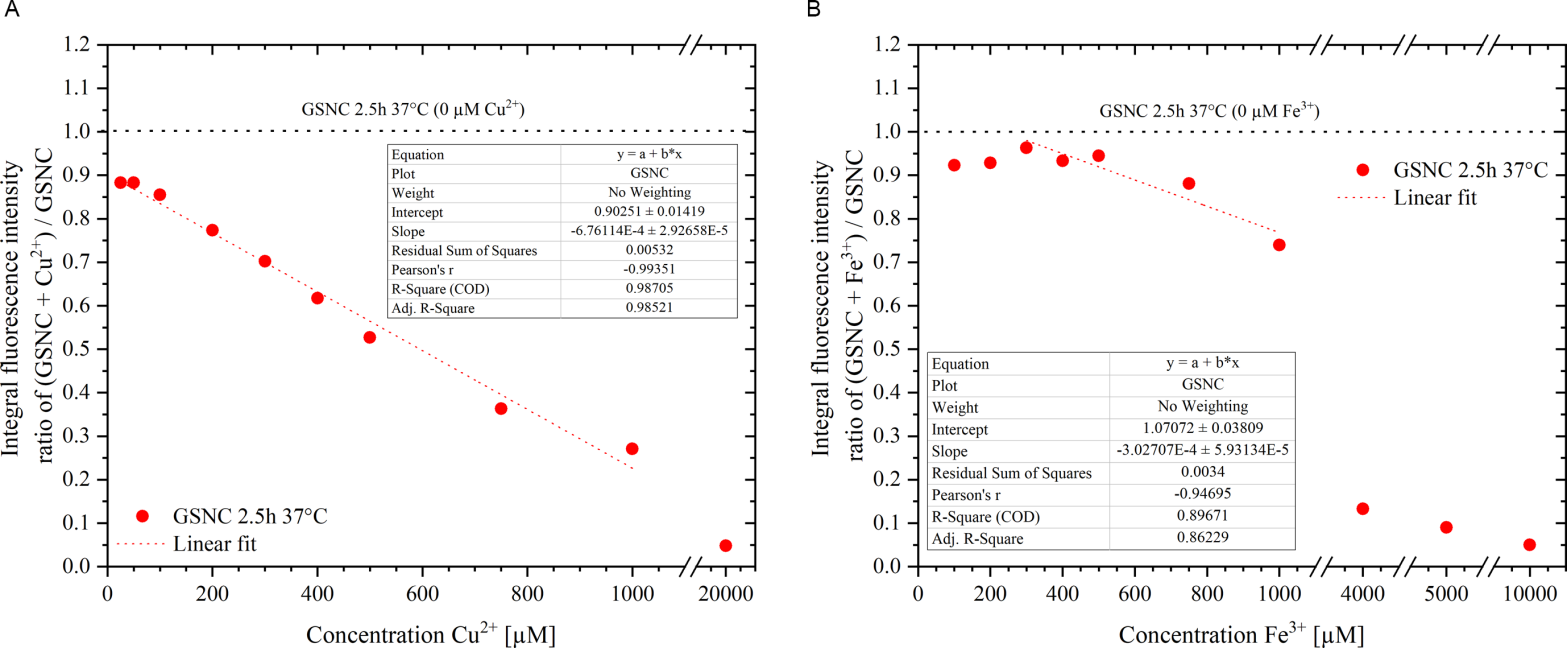
Ratios of fluorescence integral intensity of GSNC 2.5 h 37°C sample with versus without the selected metal cations as a function of metal cation concentrations (within the final systems): (*A*) Cu(II) and (*B*) Fe(III). Parameters of fluorescence measurements: excitation, 460 nm; emission range, 500–850 nm; slits, 2.5 nm; data interval, 1 nm; scan speed, 100 nm min^−1^. The obtained experimental data points are linearly fitted within the specific regions where appropriate. The details about fitting are provided as insets.

With respect to the potential application of GSNCs in optical imaging (*in vitro* as well as *in vivo*; e.g [23]), the luminescence intensity changes of GSNC 2.5 h 37°C nanocomposite (revealing the highest Au−Ag synergism) are measured in an excess of two selected transition metal ions that are crucial cofactors in living systems, Cu(II) and Fe(III) (electronic supplementary material, figure SI-8, and figure 6). Intentionally, the cations of a noble metal and a common metal were chosen (chlorides as counter ions in both cases) for their different chemical reactivity and, consequently, potential binding preferences. The abundant concentrations (tens/hundreds of µM metal cation concentrations in the final systems) were chosen according to the known levels of Cu(II) [33] and Fe(III) [34] in blood serum. Interestingly, 100 µM Cu(II) has been used recently in logic gates made of luminescent AuNCs [35]; while Fe(III) in 0−80 µM concentrations were detected *in vitro* by lipoic acid-based Au–Ag nanoclusters [36] and/or in 5–1000 µM concentration range by a combination of BSA-protected Au–Ag nanoclusters and His-stabilized AuNCs [37]. Therefore, it is important to test the fluorescence changes of our nanocomposites in the presence of Cu(II) and/or Fe(III) in 0–20 mM concentrations.

GSNC luminescence is quenched immediately after the addition of an abundance of metallic ions in both cases (Cu(II) and/or Fe(III)) and stays virtually constant within the next half an hour as demonstrated in electronic supplementary material, figure SI-8. The same behaviour was observed for AuNCs 2.5 h 37°C nanocomposite with the two selected metallic ions added, as can be seen in electronic supplementary material, figure SI-8. A list of the tested Cu(II) and Fe(III) final concentrations is given in electronic supplementary material, table SI-4, together with the observation of solution transparency and measured pH values. The latter is particularly important since the protein template surrounding GSNCs is heavily affected by pH changes (namely the protein secondary structure). Therefore, it is not surprising that around pH values of 5.3−6.3, the systems start to precipitate. Consequently, the solutions become turbid, losing their transparency and hence the possibility to be characterized by fluorescence measurements. Therefore, there are gaps within the datasets in figure 6. Importantly, the fluorescence is quenched almost linearly with increasing Cu(II) concentration until 1 mM final concentration (figure 6A). On the contrary, the fluorescence quenching is less pronounced in the presence of ferric ions in the same range of final metal cation concentrations (figure 6B). The slopes derived by linear fitting of data points within the specified regions where the linearity is obvious proved a more significant effect of Cu(II) than Fe(III) as a GSNC fluorescence quencher. The same trend has been derived for AuNCs (electronic supplementary material, figure SI-9).

As summarized in the very recent review of Farkhani *et al*. [27], there are several mechanisms of AuNC luminescence quenching, such as static quenching, photoinduced electron transfer, Förster resonance energy transfer, aggregation or inner filter effect. From all these mechanisms, in our case, precipitation takes place at several concentrations of the two selected metallic ions, obviously due to pH changes (see electronic supplementary material, table SI-4). As for the other Cu(II) and/or Fe(III) concentrations, hydrodynamic diameter and surface charges of nanocomposites increase and change significantly as evidenced by DLS and zeta potential measurements, respectively (see electronic supplementary material, tables SI-5 and SI-6, for GSNCs + selected metal cations and electronic supplementary material, tables SI-7 and SI-8, for AuNCs + selected metal cations). This further supports metallic ion-induced aggregation observed by many other authors employing Cu(II) and/or Fe(III) ions in conjunction with Au and/or Au–Ag nanoclusters [35–37].

The metallic ion-induced aggregation of our nanocomposites may be a result of the coordination of Cu(II) and/or Fe(III) with carboxyl groups and/or histidine (His) residues of the protein matrix. Considering the neutral pH of our GSNC solution (namely at 100−200 µM metal ion concentrations; electronic supplementary material, table SI-4), it can be assumed that copper ions will preferentially bind to N-terminal site (NTS) in BSA as known from the literature [38]. Recently, we have investigated the interaction of Fe(III) with BSA and have found that Fe(III) ions appear capable of interacting with NTS in BSA as well [39]. Therefore, both metal cations could be attached to the same binding site within BSA structure of GSNCs. There are, however, other possibilities of metal cations binding to BSA, like for instance to multimetal binding site (MBS) [39]. MBS consists of His105, His145 and His246 that are suggested to interact with Cu(II) and other toxic transition metals (e.g. Ni(II), Cd(II) and Zn(II)) with involvement of one or more carboxylate ligands (Asp248, Asp107 or Asp108) [40–46]. This might explain why Cu(II) is a more efficient quencher of GSNCs than Fe(III). Indeed, we hypothesize that probably Cu(II) and Fe(III) hexaaqua complexes differ in their abilities to interact with NTS and MBS within our nanocomposites. This may provide a clue for the observed differences in the extent of GSNC fluorescence quenching. Further experiments and exploitation of more sophisticated experimental techniques could shed light on this phenomenon because the exact underlying mechanism is currently unclear. However, it is beyond the scope of this study.

## 3. Conclusions

In this work, maturing conditions representing a new factor that can help to adjust Au–Ag synergism are introduced and investigated. Maturing is defined here as the procedure between the synthesis (finished by MW irradiation) and the dialysis (that stops the nanostructure evolution). It is well documented that this factor has been overlooked, underestimated, not considered or investigated intentionally so far. Au–Ag synergism is evaluated as the ratio of FQY values of GSNCs versus AuNCs. Bimetallic (GSNCs) and monometallic (AuNCs) nanocomposites were synthesized using MW irradiation and exploiting protein as a template to restrict the growth, so that luminescent nanoclusters and not plasmonic particles are formed. The protein treated in the same way (during the synthesis and maturing procedure) as the metallic nanocomposites served as a referent system. The selected variations of maturing conditions included either time (0 h, 1.25 h, 2.5 h) or temperature (room temperature, 37°C, 50°C). It was revealed that maturing time plays a significant role leading to an increase of Au–Ag synergistic effect. Based on the increasing Au–Ag synergism, the GSNC samples can be arranged as follows: 0 h RT < 1.25 h RT < 2.5 h 50°C < 2.5 h RT < 2.5 h 37°C. Regarding nanocluster size increase and PSD broadening, both evidenced by STEM (for metallic parts only) and DLS (hydrodynamic diameter of the whole nanocomposite in aqueous solution), the nanocomposites can be arranged into the order: 0 h RT < 2.5 h RT < 2.5 h 50°C. This order does not correlate with the observed increase of Au–Ag synergistic effect. In fact, a temperature of 50°C held for 2.5 h influences the secondary structure of the protein matrix significantly. Therefore, besides the increasing nanocluster size and PSD broadening during GSNC maturing, other factors, such as changes of the protein secondary structure for instance, play a key role in Au–Ag synergism as well. The results thus imply that maturing conditions of GSNC samples represent a very important factor leading to a further fine tuning of their luminescent properties, hypothetically by the different structural arrangement of atoms within Au–Ag nanoclusters. The stability of the GSNC luminescent signal was further investigated in the presence of Cu(II) and/or Fe(III) because these two selected metal cations are relevant cofactors in living systems. The observed differences in the extent of luminescence quenching caused by Cu(II) and/or Fe(III) in the concentration range of 0−20 mM were discussed and a plausible reason for such a phenomenon was suggested.

## 4. Methods

### 4.1. Chemicals

BSA (>98%), gold(III) chloride trihydrate (HAuCl_4_·3H_2_O; ≥99.9%), silver nitrate (AgNO_3_; 99.9999%), iron(III) chloride hexahydrate (FeCl_3_·6H_2_O; ≥99%), copper(II) chloride dihydrate (CuCl_2_·2H_2_O) and sodium hydroxide (NaOH; ≥98.0%) were purchased from Sigma-Aldrich (St Louis, MO, USA) and used as received (without any further purification). Hydrochloric acid (35%) was purchased from Penta s.r.o. (Prague, Czech Republic). Deionized (DI) water prepared by purging Milli-Q purified water (Millipore Corp., Bedford, MA, USA) was used for GSNC synthesis and other experiments except for ICP-MS. Nitric acid (69%, Analpure), hydrochloric acid (36%, Analpure), single element certified reference materials, aqueous calibration standard solution, ASTASOL^®^ of Au, Ag (1000.0 ± 2.0 mg l^−1^), and INT MIX 1 (10 mg l^−1^) were purchased from Analytika Ltd, Prague, Czech Republic. Ultrapure 18.2 MΩ cm water was prepared using a Milli-Q purification system (Millipore Corp., Molsheim, France) and used only for ICP-MS analyses.

### 4.2. Synthesis, maturing and dialysis of gold–silver nanocluster, gold nanocluster and BSA-T samples

The GSNC samples were synthesized in a beaker placed on a magnetic stirrer. HAuCl_4_·3H_2_O (10 mM, 800 µl) was mixed with BSA solution (66.43 mg ml^−1^) in the beaker. After 90 s, AgNO_3_ solution (8 mM, 200 µl) was added. Then, after another 90 s, NaOH (1 M, 200 µl) was added to adjust the pH above 12. Finally, after another 90 s of stirring, the beaker with the sample was placed into a microwave oven (max. power 700 W) to be heated by microwave irradiation at a power of 140 W for 10 s. Three types of GSNC samples differing in the maturing conditions (0 min and/or 2.5 h after MW irradiation, room temperature and/or 50°C) were prepared. GSNC 0 min RT sample was cooled in an ice bath immediately after MW irradiation and subsequently dialysed. GSNC 1.25 h RT and GSNC 2.5 h RT samples were allowed to develop at room temperature for 1.25 and 2.5 h, respectively. GSNC 2.5 h 37°C and GSNC 2.5 h 50°C samples were matured in a dry bath incubator at 37°C and/or 50°C for 2.5 h, respectively. Two references were synthesized for each sample: mono-metallic and non-metallic. The first reference containing only gold (AuNCs) was prepared in the same way as for GSNC samples, but instead of AgNO_3_ solution, 200 µl of DI water was added. The metal-free (non-metallic) reference, BSA-T, was synthesized using hydrochloric acid (40 mM, 800 µl) instead of gold chloride trihydrate.

All samples and references were dialysed with a 14 kDa cut-off dialysis membrane (regenerated cellulose, Membra-Cel™) against DI water. Dialysis was performed for 22 h at room temperature (22°C). The DI water was changed two times after 1 h then after another 1 h. After the dialysis, the final sample was 1.5 times diluted when compared with the volume reached immediately after the synthesis. All samples and references were synthesized in triplicate and finally mixed to get an average sample. The triplicates were prepared three times independently to verify reproducibility.

### 4.3. Gold–silver nanocluster and gold nanocluster luminescence quenching induced by Cu(II) and/or Fe(III)

The samples (GSNC/AuNCs 2.5 h 37°C) were mixed (on vortex) with aqueous solutions of the selected metal cations (CuCl_2_·2H_2_O; FeCl_3_·6H_2_O) in a volume ratio 4 : 1, i.e. the used concentrations of metal cations (M) were five times higher than the final concentrations in GSNC/AuNCs + M systems. The final BSA concentration was 16 mg ml^−1^ (240 µM). The DLS and fluorescence measurements were conducted using the same parameters as for the samples without Cu(II) and/or Fe(III).

### 4.4. Inductively coupled plasma mass spectrometry

Before ICP-MS analysis, 500 µl GSNC aliquots were dried in a vacuum rotary evaporator to dryness. The total silver and gold levels were accurately determined using a validated ICP-MS method [47]. Briefly, the dried aliquots were digested using a digestion mixture of 2 ml of concentrated nitric acid and 2 ml of concentrated hydrochloric acid in an MLS 1200 mega closed vessel microwave digestion unit (Milestone, Italy) according to the power-controlled digestion programme. The digests were allowed to cool down to laboratory temperature and diluted with ultrapure water in 25 ml volumetric flasks. The total silver and gold levels were acquired by an Agilent 7700x ICP-MS (Agilent Technologies Ltd, Japan) using seven-point external calibration within a concentration of 10–2000 µg l^−1^ for Ag and 100–10 000 µg l^−1^ for Au. The quality control sample at a concentration level of 500 µg l^−1^ for Ag and 5000 µg l^−1^ for Au was regularly analysed every ten samples to ensure the quality of the acquired results. All ICP-MS measurements were performed in six replicates, and the results are expressed as an average ± s.d.

### 4.5. Circular dichroism

CD spectra were obtained using a JASCO J-815 (Jasco, Tokyo, Japan) CD spectrometer in 0.1 cm quartz cuvettes with data interval of 1 nm, five accumulations, scan speed of 20 nm min^−1^, in the range of 200−260 nm. Average samples were measured three times and subsequently the three spectra were averaged. CD spectra were analysed in BeStSel using the following parameters: concentration, 402.6 µM; number of residues, 583; pathlength, 0.1 cm; scale factor, 80.

### 4.6. Absorbance and fluorescence measurement and quantum yield determination

Absorbance was recorded on a Specord 250 Plus-223G1032 (Analytik Jena, Jena, Germany) with a double beam arrangement using a 1 cm quartz cuvette, 2 nm slits, data interval of 1 and in the range of 250−600 nm. Fluorescence measurements of samples were performed on a JASCO F8500 (Jasco, Tokyo, Japan) spectrofluorometer in a 1 cm quartz cuvette with 2.5 nm slits, data interval of 1 nm and scan speed of 100 nm min^−1^. Emission spectra were measured in the range of 500−850 nm using 460 nm excitation. All spectra were corrected to avoid any deviations induced by instrumental components. Average samples were measured three times and subsequently the spectra were averaged.

The FQY was calculated by the following equation:


$$\phi =\phi_{S}\cdot \frac{F\cdot \left(1-10^{-A_{S}}\right)\cdot n^{2}}{F_{S}\cdot \left(1-10^{-A}\right)\cdot n_{S}^{2}},$$

where *F* is the integrated fluorescence intensity, *A* is the absorbance, *n* is the index of refraction and subscript *S* indicates the standard. DCM, 4-(dicyanomethylene)-2-methyl-6-(4-dimethylaminostyryl)-4*H*-pyran, dissolved in ethanol (99.8%, Lach-Ner, Neratovice, Czech Republic) was used as a standard (*F*_*S*_ = 0.437 ± 0.024) [48].

### 4.7. X-ray photoelectron spectroscopy

The XPS measurements of most samples were carried out with a PHI 5000 VersaProbe II XPS system (Physical Electronics) with a monochromatic Al-K_α_ source (15 kV, 50 W) and a photon energy of 1486.7 eV. All the spectra were measured in a vacuum of 1.1 × 10^–7^ Pa and at a room temperature of 20°C. Dual beam charge compensation was used for all measurements. The spectra were evaluated with MultiPak software, version 9 (Ulvac-PHI, Inc., Chanhassen, MN, USA).

The sample labelled as GSNC 0 min RT was analysed with a Nexsa G2 XPS system (Thermo Fisher Scientific) also equipped with a monochromatic Al-K_α_ source and photon energy of 1486.7 eV. The spectral acquisition was maintained at 1.6 × 10^–9^ mBar, and at a controlled ambient temperature of 20°C. Charge neutralization techniques were systematically employed throughout the measurement. Spectral analysis and data interpretation were conducted using Avantage software version 6.5.1 provided by Thermo Fisher Scientific.

### 4.8. Dynamic light scattering and zeta potential measurement

Hydrodynamic diameter and zeta potential of samples were determined using a Zetasizer Nano ZEN 3600 ZS (Malvern Instrument Ltd, Malvern, UK) equipped with a He–Ne laser. Average samples were measured at 22°C. Values in the table and plotted graphs are the average of 10 measurements. The s.d. values were also determined.

### 4.9. STEM, HR-TEM and EDS

All three GSNC samples (final protein concentration of 0.3 mg ml^−1^) were drop-cast (2–4 µl) on glow discharged TEM copper grids covered with lacey carbon and allowed to dry spontaneously at room temperature. Then, the samples were measured by HR-TEM Titan G2 60-300 (FEI, Hillsboro, OR, USA) with an image corrector with an accelerating voltage of 300 kV. Images were taken with a BM UltraScan CCD camera (Gatan, Pleasanton, CA, USA). EDS was performed in STEM mode by a Super-X system with four silicon drift detectors (Bruker, Billerica, MA, USA). STEM images were taken with an HAADF detector 3000 (Fishione, Export, PA, USA). HAADF mode of STEM was intentionally used to better visualize NCs because heavier elements appear bright, while lighter elements appear dark.

## Data Availability

The datasets supporting this article are available as part of the electronic supplementary material [49]. The raw/processed data required to reproduce these findings can be requested from the authors. They are also available in the national repository CZ (https://data.narodni-repozitar.cz/) [50].
